# To Our Readers

**Published:** 1889-10

**Authors:** 


To Our Readers.—The year is drawing to a close, the crops are good, and the times are prosperous, and yet many of our readers have not paid their subscriptions. Friends, it takes a great deal of labor and money to send you the Record month after month, laden with interesting and useful matter for the busy practitioner, and the printers require hard cash at our hands. Can you not devote the proceeds of a single professional visit to paying the editor? Suppose you try it between now and our next issue. If you will you will make glad the hearts of the editors, and we guarantee that you will feel the better for it.

